# An mRNA influenza vaccine induces immunity comparable to an adjuvanted vaccine in a randomized trial

**DOI:** 10.1038/s41541-026-01370-7

**Published:** 2026-01-14

**Authors:** Carole Henry, Daniel Makrinos, Runxia Liu, Maria Cavallaro, Brooke Fenderson, Yanbo Sun, Xiaolin Chang, Eleanor Astley, Bethany Girard, Wen-Han Yu, Jaap Oostendorp, Anthony DiPiazza, Robert Paris

**Affiliations:** 1 Moderna, Inc., Cambridge, MA USA; 2 GSK, Cambridge, MA USA; 3 Dartmouth College, Hanover, NH USA; 4 Tufts Medical Center, Boston, MA USA

**Keywords:** Diseases, Immunology, Microbiology

## Abstract

Influenza causes substantial morbidity and mortality worldwide. This randomized, open-label, phase 1 trial (ClinicalTrials.gov, NCT05397223, date of registration: May 31, 2022) compared the immunogenicity of an mRNA-based quadrivalent influenza hemagglutinin (HA) vaccine (mRNA-1010) with a licensed comparator (FLUAD) in adults aged 18-75 years. We evaluated humoral and cellular immune responses using hemagglutination inhibition assays, flow cytometry-based memory B cell (MBC) profiling, and intracellular cytokine staining for T-cell characterization. Both vaccines elicited durable hemagglutination inhibition titers and increased HA-specific MBC responses across four vaccine strains. Compared with FLUAD, mRNA-1010 induced higher frequencies of classical and activated MBCs specific to the H3 HA included in the vaccine, while inducing similar MBC responses to the other strains. mRNA-1010 and FLUAD generated strong HA-specific CD4^+^ T-cell responses; a trend toward higher CD8^+^ T-cell responses was observed in mRNA-1010 recipients compared with FLUAD recipients for two of the four strains. These findings support the potential of the mRNA platform for seasonal influenza vaccination.

## Introduction

Each year, nearly a billion cases of influenza virus infections are estimated to occur worldwide, resulting in approximately five million severe cases and over half a million deaths^1,2^. Although vaccines are recommended for high-risk groups, including pregnant women, young children, and older adults, influenza continues to cause significant illness and deaths annually, highlighting the urgent need for next-generation influenza vaccines that offer more effective protection against disease^3^.

Traditional influenza virus vaccines are produced in eggs; however, mutations can arise that affect antigenicity and reduce vaccine efficacy^4,5^. Novel vaccine platforms, such as recombinant hemagglutinin vaccine produced in insect cells, or subunit inactivated vaccine derived from virus propagated in mammalian cells, have been developed and are now approved^6,7^. For the older adult population, the use of adjuvanted influenza virus vaccines has been shown to confer enhanced protection^8^. More specifically, it has been demonstrated that the adjuvant MF59, present in the vaccine FLUAD, has the ability to induce priming of influenza antigen-specific CD4^+^ T-cell responses, to induce strong memory T- and B-cell responses, and to broaden the immune response beyond the influenza strains included in the vaccine^9^.

In recent years, mRNA vaccines have gained significant attention, particularly during the COVID-19 pandemic. They induce strong immunogenicity across age groups, have a favorable safety profile, can incorporate multiple antigens, and have the flexibility to rapidly update viral sequences^10^. We have developed an mRNA-based seasonal influenza hemagglutinin (HA) vaccine, mRNA-1010, that was demonstrated to be safe and immunogenic in healthy adults^11^. There are currently no studies directly comparing the durability of immune responses induced by mRNA-based influenza vaccines and adjuvanted influenza virus vaccines. To answer this question, we compared the humoral and cellular immune responses induced by our influenza HA-specific mRNA vaccine, mRNA-1010, with those of a licensed adjuvanted influenza virus vaccine, FLUAD, across sex- and age-matched individuals 18–75 years of age.

## Results

### mRNA-1010 and FLUAD elicit durable HAI responses after vaccination

**Fig. 1:**
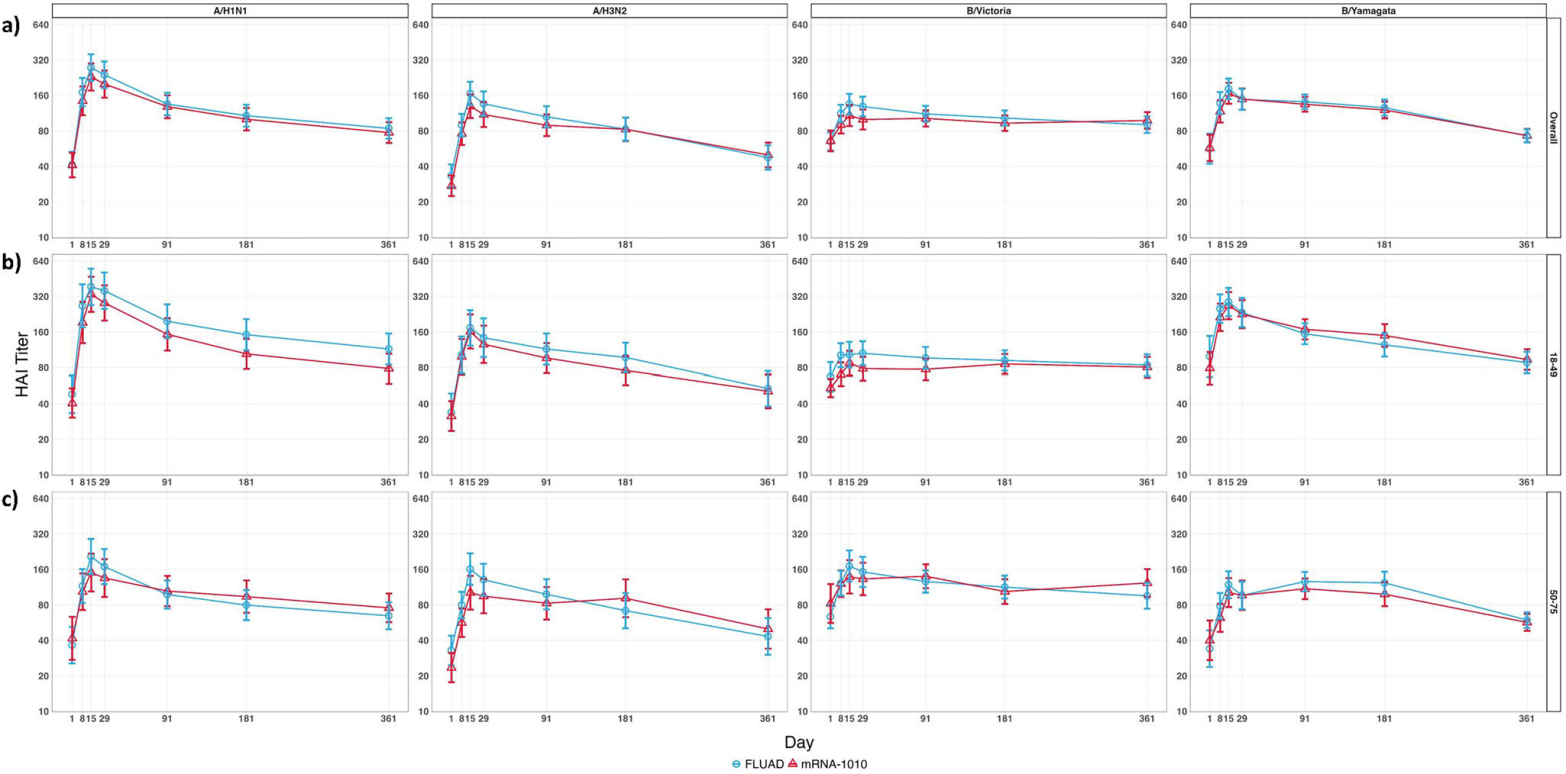
mRNA-1010 and FLUAD vaccination elicited durable HAI responses. GMTs with 95% CI of anti-HA antibody levels following mRNA-1010 (purple) and FLUAD (green) at days 1, 8, 15, 29, 91, 181, and 361 are shown (**a**) for all participants, (**b**) for younger adults 18–49 years of age (mRNA-1010: *n* = 23–29; FLUAD: *n* = 21–27 for each time point), and (**c**) for older adults 50–75 years of age (mRNA-1010: *n* = 21–26; FLUAD: *n* = 24–30 for each time point). Antibody values reported as below the LLOQ are replaced by 0.5 × LLOQ. Values greater than the ULOQ are converted to the ULOQ. For baseline (day 1), GMTs with 95% CIs were estimated using a *t*-test. For post-baseline time points, GMTs with 95% CIs for mRNA-1010 and FLUAD groups were estimated based on a separate mixed model for repeated measures, with baseline log10 titer, vaccination group, day, and vaccination group by day interaction as fixed effects and participants as a random effect. Influenza A H1N1 antibody (titer): LLOQ: 10, ULOQ: 1280 from day 1 to day 29, and LLOQ: 10, ULOQ: 4305 from day 91 to day 361; influenza A H3N2 antibody (titer): LLOQ: 10, ULOQ: 2560 from day 1 to day 29, and LLOQ: 10, ULOQ: 4305 from day 91 to day 361; influenza B/Victoria-lineage (titer): LLOQ: 10, ULOQ: 640 from day 1 to day 29, and LLOQ: 10, ULOQ: 4561 from day 91 to day 361; influenza B/Yamagata-lineage (titer): LLOQ: 10, ULOQ: 2560 from day 1 to day 29, and LLOQ: 10, ULOQ: 5120 from day 91 to day 361). A-H1N1, A/Wisconsin/588/2019 (H1N1) pdm09-like virus; A-H3N2, A/Darwin/6/2021 (H3N2)-like virus; B/Vict, B/Austria/1359417/2021 (B/Victoria lineage)-like virus; B/Yam, B/Phuket/3073/2013 (B/Yamagata lineage)-like virus; CI confidence interval, GMT geometric mean titer, HA hemagglutinin, LLOQ lower limit of quantification, ULOQ upper limit of quantification.

As reported previously, post-vaccination influenza hemagglutination inhibition (HAI) geometric mean titers (GMTs) and geometric mean fold rises (GMFRs) at days 8, 15, and 29 were comparable following a single injection of mRNA-1010 or FLUAD across all four vaccine-matched strains (A-H1N1, A-H3N2, B/Victoria, B/Yamagata)^12^. Here, we report HAI titers measured at day 91, day 181, and up to day 361 post-vaccination to assess the durability of antibody responses induced by mRNA-1010 in comparison with FLUAD. Robust HAI antibody titers were maintained up to day 181. Notably, the waning of the HAI antibody response over time was similar between mRNA-1010 and FLUAD for all four strains assessed and in both age groups (18–49 and 50–75 years) (Fig. 1). Overall, GMTs (Fig. 1a), GMFRs, seroresponse rates, and percentage of participants with HAI titers ≥1:40 (Supplementary Table 1) were similar following a single dose of mRNA-1010 or FLUAD vaccine at the day 91, day 181, and day 361 time points. While peak titers (days 15 and 29) were generally higher in the younger cohort (18–49 years of age) compared with the older cohort (50–75 years of age), there was no clear difference between both age groups at the day 181 and day 361 time points (Fig. 1b, c; Supplementary Tables 2 and 3). Overall, HAI antibody titers remained above baseline for all four influenza strains at day 361 in both the mRNA-1010 and FLUAD groups (Supplementary Table 1).

### Robust induction of HA-specific memory B cell responses by mRNA-1010 and FLUAD

**Fig. 2:**
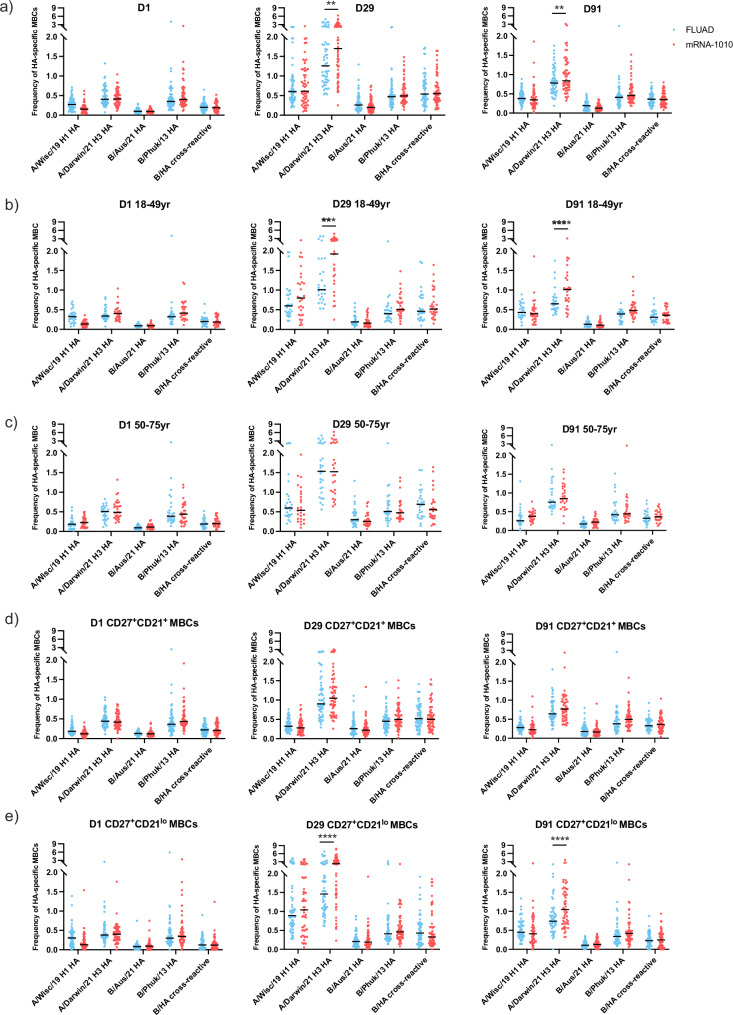
mRNA-1010 vaccination induced higher frequency of H3 HA-specific memory B cells compared with FLUAD while inducing similar MBC responses to other HAs present in the vaccines. The frequency of antigen-specific MBCs following FLUAD (in blue, *n* = 53–56 depending on time point) and mRNA-1010 (in red, *n* = 54–55 depending on time point) against A/Wisconsin/588/2019 H1 HA, A/Darwin/6/2021 H3 HA, B/Austria/1359417/2021 HA, and B/Phuket/3073/2013 HA are shown (**a**) on days 1, 29, and 91 for all participants, (**b**) on days 1, 29, and 91 for younger adults (18–49 years old), (**c**) on days 1, 29, and 91 for older adults (50–75 years old), (**d**) on days 1, 29, and 91 looking at classical CD27^+^CD21^+^ MBCs, and (**e**) on days 1, 29, and 91 looking at activated CD27^+^CD21^lo^ MBCs for all participants. A two-way ANOVA test was used to test for differences between groups (*p < 0.05, **p < 0.005, ***p < 0.0005, ****p < 0.0001). ANOVA analysis of variance, HA hemagglutinin, MBC memory B cell.

Given the robust induction of antibody responses across the vaccine groups and age cohorts, we next aimed to profile the magnitude and kinetics of HA-specific memory B cells (MBCs) over time, from day 1 to day 91 (Supplementary Fig. 2). At baseline (day 1), no differences were observed between the FLUAD and mRNA-1010 groups in the frequency of antigen-specific CD27^+^ MBCs across all four vaccine-matched strains. At days 29 and 91, similar frequencies of HA-specific CD27^+^ MBCs were observed across both the FLUAD and mRNA-1010 groups against the H1 A/Wisconsin/588/2019, B/Phuket/3073/2013, and B/Austria/1359417/2021 strains. In contrast, mRNA-1010 induced higher frequencies of H3 A/Darwin/6/2021 HA-specific CD27^+^ MBCs at both time points (Fig. 2a), in younger adults but not in older adults (Fig. 2b, c). Because the downregulation of CD21 expression is associated with activation of MBCs post-vaccination^13,14^, we further dissected antigen-specific MBC populations based on this surface marker. When comparing CD27^+^CD21^+^ (classical) MBCs versus CD27^+^CD21^lo^ (activated) MBCs, the increase in frequency of H3 A/Darwin/6/2021 HA-specific MBCs observed in participants (all ages) administered mRNA-1010 at day 29 and day 91 was driven by an expansion of the CD27^+^CD21^lo^ (activated) MBCs (Fig. 2d, e), known to be a population of recent germinal center (GC) graduates primed for plasma cell differentiation^15^. In conclusion, similar antigen-specific MBC frequencies were induced following vaccination with either mRNA-1010 or FLUAD, except for the H3N2 HA, where mRNA-1010 induced more robust activated MBCs to that antigen, suggesting a potential enhanced GC activity.

### mRNA-1010 vaccination elicits HA-specific CD4^+^ and CD8^+^ polyfunctional T cells

**Fig. 3:**
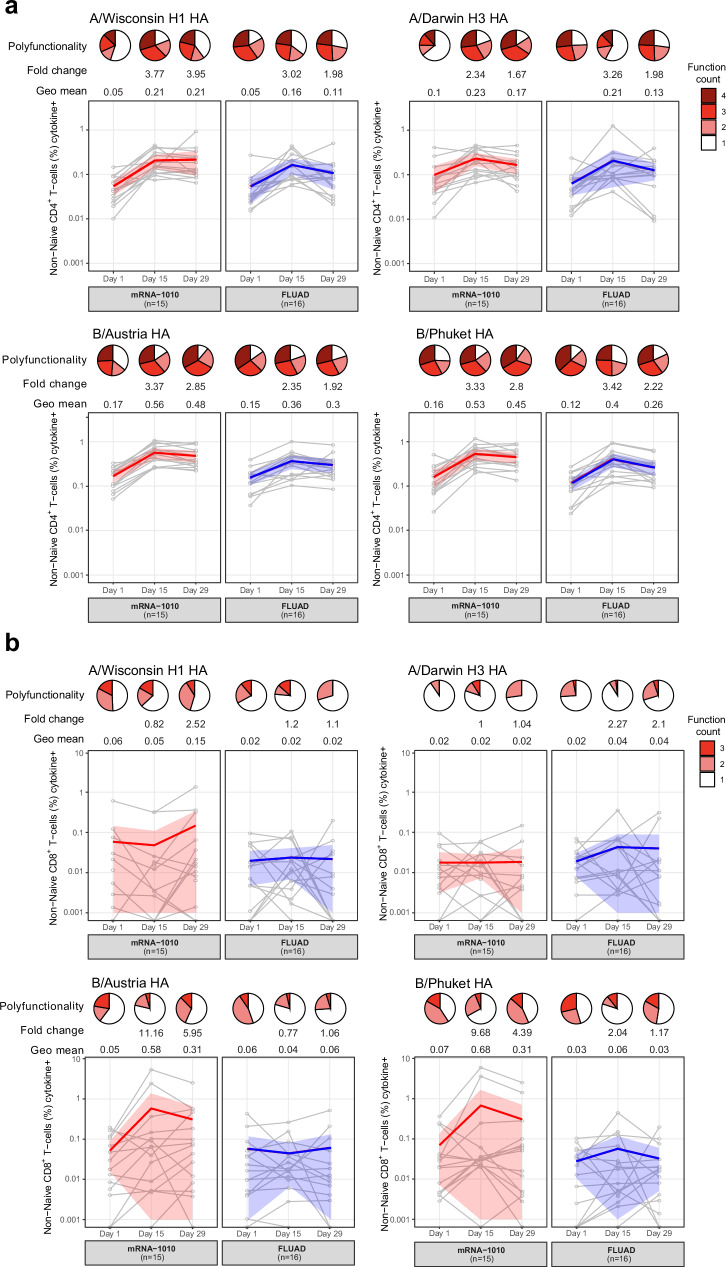
mRNA-1010 and FLUAD boost baseline HA-specific CD4^+^ T-cell responses. Antigen-specific T-cell responses against A/Wisconsin/588/2019 H1 HA, A/Darwin/6/2021 H3 HA, B/Austria/1359417/2021 HA, and B/Phuket/3073/2013 HA following mRNA-1010 (*n* = 15) or FLUAD (*n* = 16) vaccination. PBMC samples were collected on days 1, 15, and 29, and antigen-specific CD4^+^ Th1 (**a**) and CD8^+^ (**b**) T-cell responses were assessed using an ICS assay. Antigen-specific CD4^+^ Th1 T cells are defined as CD4^+^ T cells positive for at least one of the following markers: IFN-γ, TNF-α, IL-2, and CD40L. Antigen-specific CD8^+^ T cells are defined as CD8^+^ T cells positive for at least 1 of the following markers: IFN-γ, TNF-α, IL-2. Each gray line represents an individual participant. The bold lines represent the geometric mean for each group: mRNA-1010 (in red) and FLUAD (in blue). The shaded areas around the bold lines indicate the 95% confidence intervals of the group means. The pie charts represent polyfunctionality as the fraction of T cells expressing respective number of markers as indicated in the figure legend calculated from the arithmetic mean. Significant differences between mRNA-1010 and FLUAD were observed using a two-way ANOVA with Šídák’s multiple comparisons test on days 15 and 29 for B/Austria (*p* = 0.0203 and *p* = 0.0438, respectively), and on day 29 for B/Phuket (*p* = 0.0298). ANOVA analysis of variance, HA hemagglutinin, ICS intracellular cytokine staining, IFN interferon, IL interleukin, PBMC peripheral blood mononuclear cell, TNF tumor necrosis factor.

Next, we sought to profile the magnitude and kinetics of HA-specific CD4^+^ T helper (Th) 1 and CD8^+^ T-cell responses in a subset of vaccine recipients (mRNA-1010, *n* = 15 [one subject excluded for failing to meet assay criteria]; FLUAD, *n* = 16) on days 1, 15, and 29 using a T-cell intracellular cytokine staining assay (Supplementary Fig. 3). Following a single injection of either mRNA-1010 or FLUAD, HA-specific CD4^+^ Th1 T-cell responses were significantly enhanced, peaking on day 15 and remaining elevated through day 29 across all assessed strains in both age groups. The kinetics and magnitude of HA-specific CD4^+^ T-cell responses were very similar when comparing the mRNA-1010 to FLUAD groups (Fig. 3a). Although CD8^+^ T-cell responses were less pronounced compared with CD4^+^ Th1 T-cell responses, they were still detectable. The kinetics of CD8^+^ T-cell responses were overall similar following vaccination with either mRNA-1010 or FLUAD. While mRNA-1010 and FLUAD induced similar magnitude of responses to the A/Darwin/6/2021 (H3N2) HA, responses were generally higher following mRNA-1010 vaccination for the influenza A H1N1 strain and influenza B strains (both B/Austria and B/Phuket HAs) compared with FLUAD (Fig. 3b). Polyfunctional CD8^+^ T cells were defined as those expressing interferon-γ (IFN-γ), tumor necrosis factor- α (TNF-α), and/or interleukin (IL)-2, while CD4^+^ Th1 T cells were assessed for IFN-γ, TNF-α, IL-2, and/or CD40L. Interestingly, both vaccines induced a high level of polyfunctionality for both CD4^+^ Th1 and CD8^+^ T-cell responses, indicated by a high percentage of HA-specific CD4^+^ Th1 or CD8^+^ T cells being positive for more than one cytokine (pie charts in Fig. 3a, b). HA-specific CD4^+^ Th2 T-cell responses were also measured (IL-4, IL-5, and IL-13 cytokines). Overall, Th2 responses were markedly lower in magnitude compared with Th1 responses, consistent with observations from other mRNA vaccines (Supplementary Fig. 4)^12,16–18^. For the influenza A strains (H1N1 and H3N2), IL-4 and IL-13 responses were near the limit of detection, and IL-5 responses were minimal post-vaccination across all vaccine groups (Supplementary Fig. 4c). While CD4^+^ Th2 T-cell responses to influenza B HAs were marginally higher than influenza A HA responses post-vaccination, the frequency of IL-5 was more than 10-fold lower than the frequency of IFN-γ. In conclusion, comparable HA-specific CD4^+^ Th1, Th2, and CD8^+^ T-cell response kinetics were observed between the mRNA-1010 and FLUAD groups. The overall magnitude of HA-specific CD4^+^ Th1 T-cell responses was similar between both vaccine groups, while there was a trend for mRNA-1010 to induce higher CD8^+^ T-cell responses for three of the four strains present in the vaccine. The CD4^+^ T-cell responses to both mRNA-1010 and FLUAD were Th1 biased, with low levels of Th2 cytokines being detected (Fig. 3 and Supplementary Fig. 4).

## Discussion

mRNA-based vaccines have garnered substantial interest, particularly following the COVID-19 pandemic. Here, we sought to compare the humoral and cellular immune responses of an mRNA-based quadrivalent influenza HA vaccine, mRNA-1010, to an FDA-approved adjuvanted inactivated influenza virus vaccine, FLUAD. The safety and immunogenicity (up to day 29) from this phase 1 trial have been reported recently^12^. These previously reported safety findings demonstrated that mRNA-1010 and FLUAD were generally well-tolerated, with no treatment-related severe or serious adverse events (AEs), and no AEs of special interest^12^. Similarly, no treatment-related severe or serious AEs or AEs of special interest were observed during long-term follow-up up to day 361. Here, we focused on long-term antibody responses (up to a year after vaccination), as well as cellular responses.

We demonstrate here that mRNA-1010 induces antigen-specific humoral and cellular immune responses that were overall comparable to immune responses induced by FLUAD. We observed that robust HAI antibody titers were induced with similar durability profiles (up to 361 days post-vaccination) between mRNA-1010 and FLUAD for all four strains assessed and in both younger (18–49 years of age) and older (50–75 years of age) adults. The results from the mRNA-1010 vaccinees were consistent with a prior phase 1/2 study that evaluated antibody responses up to day 181^11^. Importantly, a recently optimized mRNA-1010 candidate has been evaluated in a recent phase 3 safety and immunogenicity study, which demonstrated that mRNA-1010 elicited statistically superior immune responses relative to licensed standard- or high-dose seasonal influenza vaccines in younger and older adults^19^.

Traditional inactivated influenza virus vaccines primarily trigger an immune response against the HA glycoprotein, with a smaller contribution from neuraminidase (NA), although the amount of NA is not standardized in these vaccines^20^. Because mRNA-1010 encodes for the four seasonal HAs and does not contain any NA-encoding mRNAs, we did not measure NA responses in this study.

It is known that HA-specific antibodies generated by nonadjuvanted inactivated influenza vaccines wane over time^21,22^. This decline is more pronounced in older adults, who often experience a drop in vaccine effectiveness and seroprotective antibody levels as the flu season progresses^21,22^. In this age group, antibody levels show exponential decline and can fall to nonprotective levels within 12 months after vaccination^21,22^. FLUAD is among the flu vaccines recommended by the US Centers for Disease Control and Prevention for individuals aged 65 years and older^23^ and, in the elderly population (≥75 years), the adjuvanted vaccine was reported to be more immunogenic than the unadjuvanted vaccine^24^. FLUAD is also more immunogenic than nonadjuvanted vaccines in non-elderly adults (18–60 years) with chronic diseases^25^ and in children^26^. The HAI titers induced by mRNA-1010 at 6 months (day 181) post-vaccination in adults 50–75 years of age observed in this study were well above baseline HAI titers and above the protective threshold of 1:40 for all four strains. While our study had a limited number of participants, especially in the ≥65 years of age group, and humoral immunogenicity data are limited to strain-specific HAI antibody titers, it is encouraging that mRNA-1010 induced antibody responses that are comparable to FLUAD-induced responses, a vaccine that has shown clinical benefits compared with unadjuvanted vaccines in older adults and at-risk individuals^27^.

We then profiled antigen-specific MBCs over time in participants administered mRNA-1010 or FLUAD. We demonstrated that the overall frequencies of HA-specific MBCs were comparable between the mRNA-1010 and FLUAD vaccine groups up to day 91. Interestingly, among younger adults (18–49 years of age), mRNA-1010 induced higher frequencies of H3 A/Darwin/6/2021 H3 HA-specific CD27^+^ MBCs at both days 29 and 91 compared with FLUAD, mostly driven by higher frequencies of HA-specific CD27^+^CD21^lo^ (activated) MBCs in younger adults. MBCs are long-lived cells that convert rapidly into plasmablasts (also called antibody-secreting cells) upon antigen re-exposure^28^. The role of these plasmablasts is to quickly increase circulating antibody titers to protective levels. It has been demonstrated that the MBC compartment contains a broader repertoire compared to steady-state serum antibodies (coming from long-lived plasma cells)^29^, highlighting the fact that MBCs are likely important against circulating strains that drifted from vaccine antigens. Such responses are not rapid enough to prevent infection but may help protect against severe symptoms or help shorten illness duration. In addition, a human challenge study demonstrated that B and T cell repertoire diversity metrics correlated with shedding outcome, suggesting a potential role in limiting transmission^30^.

Antigenic drift is observed most frequently in the influenza A/H3N2 subtype compared with other subtypes and can cause a decrease in vaccine-induced immunogenicity^31^, highlighting the importance of generating strong immune responses against H3N2 strains. It is of interest to test this observation against future mRNA-1010 compositions with updated seasonal H3N2 strains. One minor caveat is that the HA protein used in the MBC assessment was produced in mammalian cells from the A/Darwin/6/2021 H3 sequence, which matches the mRNA-1010 sequence, while the H3 antigen in FLUAD was derived from an egg-grown virus (A/Darwin/9/2021)^32^. Because the two H3 sequences are 98% identical, we do not believe that the impact will be significant.

In a previous study in humans, it was shown that participants receiving mRNA-1010 produced more robust plasmablast responses and stimulated robust recall B-cell responses characterized by sustained GC reactions by looking at lymph-node fine needle aspirates^33^. The increase in frequency of HA-specific CD27^+^CD21^lo^ (activated) MBCs, a population known to be recent GC graduates primed for plasma cell differentiation, observed here, could be related to the increased GC activity observed following mRNA-1010 vaccination. It would be interesting in the future to assess MBC frequencies past day 91. Although we did not measure antigen-specific CD4 T follicular helper cells in this study, we did observe CD40L upregulation by CD4 T cells following mRNA-1010 vaccination, which is critical for B-cell survival and GC maintenance^34^.

Traditional inactivated influenza virus vaccines generally induce strong CD4^+^ T-cell responses but weak CD8^+^ T-cell responses^35^. In adults, a subunit inactivated vaccine (Flucelvax) elicited weak CD8^+^ responses against the various influenza antigens up to day 91 post-vaccination^36^. Flublok, a recombinant HA vaccine (higher dose than inactivated vaccines), has been shown to induce higher levels of CD4^+^ T-cell responses when compared with traditional influenza vaccines^37^, as well as increased multiple cytokine-secreting CD8^+^ T-cell responses^38^.

The two vaccine platforms assessed in this study may result in different kinetics, magnitude, and polyfunctionality of T-cell responses based on antigen expression/stability, MHC class I, and MHC class II presentation. In the context of mRNA vaccines, following uptake by antigen-presenting cells (APCs), the encoded proteins are endogenously synthesized by host cells and presented on MHC class I or MHC class II molecules or excreted^39^. In addition, RNA-expressed proteins are fragmented intracellularly, and the peptides are displayed on MHC class I molecules, leading to antigen-specific CD8^+^ T-cell responses^20^.

We demonstrate here that, overall, the kinetics (peak at day 15) and magnitude of HA-specific CD4^+^ Th1 T-cell responses induced by mRNA-1010 and FLUAD were comparable for all four HA strains tested. Previous studies have demonstrated that adjuvanted influenza vaccines, like FLUAD, induced higher CD4^+^ T-cell responses compared with standard flu vaccines against H1N1 and H3N2 subtypes, and that the CD4^+^ T cells were polyfunctional (IL-2, or IL-2 and IFN-γ)^40^. Adjuvanted influenza vaccines have shown higher CD4^+^ Th1 T-cell responses compared with the standard dose flu vaccines^38,41^. Although the observed CD8^+^ T-cell responses were generally modest, and more variable, we also demonstrate that while FLUAD and mRNA-1010 induced similar CD8^+^ T-cell responses for influenza A HAs (H1 and H3), mRNA-1010 induced generally higher CD8^+^ T-cell responses against both influenza B HAs, at both the effector phase (day 15) and memory phase (day 29), as indicated by increased mean responses and fold changes. Although T-cell responses were not assessed past day 29 in the current study, future studies could provide further evidence of the durability of T-cell responses induced by mRNA vaccines.

It has been consistently reported that mRNA vaccines induce a predominantly Th1-polarized CD4^+^ T-cell response in adults with minimal Th2 responses, across a range of pathogens^12,16–18^. While the Th1 bias of mRNA vaccines has been well-documented^12,42^, the immunological profile induced by MF59-adjuvanted inactivated whole-virus vaccines can vary depending on the specific antigen and immunization context^38,43,44^. In our study, we observed minimal Th2-polarized CD4^+^ T-cell response in both the mRNA-1010 and FLUAD vaccine groups for all four HAs tested and throughout the study period. Although a modest increase in Th2-associated responses was detected against influenza B strains compared with A strains, the overall Th1/Th2 ratio remained greater than 10-fold, underscoring the dominant Th1-skewed response elicited by both vaccine platforms under the tested conditions.

In summary, our data comparing a first-generation mRNA vaccine with an adjuvanted, enhanced, seasonal influenza vaccine indicate that the breadth and quality of the elicited immune response are similar for the two vaccine platforms, with the exception of the H3-specific memory B-cell response, perhaps as a result of the robust germinal response observed for mRNA vaccines^33^. We acknowledge that the mRNA-1010 vaccine assessed in this study did not contain the optimizations in antigen design tested in subsequent studies in which mRNA-1010 resulted in noninferior and superior HAI responses compared with inactivated standard or high-dose influenza vaccines^19^. The data presented here, in addition to other published data, indicate that mRNA-based vaccines are a promising alternative to currently licensed influenza vaccines, with the additional advantages of the mRNA technology that can incorporate rapid strain updates and a flexible manufacturing platform that could enable the production and distribution of regionally matched influenza vaccines for improved effectiveness.

## Methods

### Randomization, samples and vaccines

In this phase 1 trial (ClinicalTrials.gov, NCT05397223, date of registration: May 31, 2022), participants were randomized 1:1 to receive a single-dose mRNA-1010 (seasonal influenza quadrivalent HA mRNA vaccine; 50 µg) or an active comparator, FLUAD (adjuvanted influenza virus vaccine; 60 µg) with an approximately equal distribution of age groups (18–49 and 50–75 years) (Supplementary Fig. 1). For participants randomized to receive mRNA-1010, the mean ages (range) for the younger and older age group were 37 (18–49) and 56 (50–68), respectively, with 3 participants ≥65 years of age. A similar age distribution was achieved in participants randomized to receive FLUAD, with a mean age (range) for the younger and older age groups of 35 (21–47) and 57 (50–73), respectively, and 5 participants ≥65 years of age. Blood samples were collected from vaccinated individuals at baseline (day 1), 7 days (day 8), 14 days (day 15), 28 days (day 29), 90 days (day 91), 180 days (day 181) and 360 days (day 361) post-vaccination as part of the mRNA-CRID-001 clinical trial (ClinicalTrials.gov, NCT05397223, date of registration: May 31, 2022). Peripheral blood mononuclear cell (PBMC) samples were prepared from whole blood specimens using standard Ficoll separation methods and were cryopreserved and stored in liquid nitrogen vapor phase until testing. Details on the study design, safety, and humoral immunogenicity data up to day 29 have been previously reported^12^. The protocol was approved by a central institutional review board (Advarra, Ref# Pro00061954). The study was conducted in accordance with the protocol, the principles of the International Council for Harmonisation Good Clinical Practice guidelines, the Declaration of Helsinki, and national, state, and local laws/regulations. All participants provided written informed consent for participation in the study, including all evaluations and study procedures specified by the protocol.

The vaccines’ composition followed the 2022/2023 seasonal influenza World Health Organization recommendation, with the difference that FLUAD followed the egg-based and mRNA-1010 the cell-based recommendation, respectively. Both H1 and H3 HAs were antigenically similar, but FLUAD included the A/Victoria/2570/2019 (H1N1) pdm09-like strain and A/Darwin/9/2021 (H3N2)-like strain, whereas the mRNA-1010 vaccine included the A/Wisconsin/588/2019 (H1N1) and A/Darwin/6/2021 (H3N2) strains. Both vaccines included the B/Austria/1359417/2021 (B/Victoria lineage) and B/Phuket/3073/2013 (B/Yamagata lineage) strains.

### Protein production and purification for memory B cell assessment

Proteins of interest were produced in-house using Expi293 cells through transfection with a plasmid vector. Optimal expression conditions were established to maximize protein yield. Post-expression proteins were purified via a combination of affinity and size exclusion chromatography (SEC). The purity and integrity of the proteins were confirmed using SEC. This process ensured high-quality proteins for downstream applications. The constructs and yields are provided in the Supplementary Table 4, and the SEC profile of each construct is shown in Supplementary Fig. 5.

### Assessment of antibody responses by hemagglutination inhibition assay

Serum samples were collected on days 1 (baseline), 8, 15, 29 (mRNA-1010: *n* = 55; FLUAD: *n* = 57), 91 (mRNA-1010: *n* = 52; FLUAD: *n* = 54), 181 (mRNA-1010: *n* = 49; FLUAD: *n* = 52), and 361 (mRNA-1010: *n* = 44; FLUAD: *n* = 45). End points included GMTs, GMFRs, relative to day 1, and seroresponse rates measured by HAI assay. Seroresponse rate was defined as the percentage of participants with a post-vaccination HAI titer ≥1:40 if baseline titer was < 1:10, or a minimum 4-fold rise in post-vaccination HAI titer if baseline titer was ≥1:10. Percentages of participants with HAI titers ≥1:40 were also assessed. Sera from participants were tested by HAI assay using standard methods^11,45^, and the strains A/Delaware/55/2019, A/Darwin/11/2021, B/Phuket/3073/2013, and B/Connecticut/01/2021. In brief, the receptor-destroying enzyme was used to remove nonspecific inhibitors of hemagglutination. Eleven two-fold serial dilutions of treated serum samples were prepared in duplicate 96-well plates. Working dilutions of influenza virus equal to four HA units per well (per World Health Organization recommendations) were added to the serum dilutions and incubated for 60 min at room temperature. Guinea pig red blood cell (RBC) suspension (0.75%) was added to the serum-virus mixture and incubated for 60 min at room temperature. Following incubation, plates were read, and the GMT was calculated from duplicate plate readings. The HAI titer was defined as the last dilution at which agglutination of red blood cells was inhibited. No statistical testing was performed for the HAI assay.

### Assessment of antigen-specific memory B cells by flow cytometry

Cryopreserved PBMC samples collected on days 1 (mRNA-1010: *n* = 54; FLUAD: *n* = 56), 29 (mRNA-1010: *n* = 55; FLUAD: *n* = 55), and 91 (mRNA-1010: *n* = 54; FLUAD: *n* = 53) were thawed and stained with monoclonal antibodies against human CD3 (BD Biosciences), CD8 (BD Biosciences), CD14 (BD Biosciences), CD56 (BD Biosciences), CD19 (Beckman Coulter), CD20 (BioLegend), CD21 (BioLegend), CD27 (BioLegend) and CD38 (BioLegend). Recombinant HA proteins of the A/Wisconsin/588/2019 (H1N1), A/Darwin/6/2021 (H3N2), B/Austria/1359417/2021, and B/Phuket/3073/2013 strains were produced in house, then biotinylated and conjugated with either Phycoerythrin (PE), Brilliant Violet 605 (BV605), Brilliant Violet 711 (BV711), or Alexa-Fluor 647 (AF647)-streptavidin, according to the manufacturer’s instruction (BioLegend), to form tetramers to bait for antigen-specific B cells. For the H1 and H3 HAs, we incorporated previously described mutations, such as *Y98F* in tandem with an N-linked glycosylation site proximal to the receptor binding site of HA to remove nonspecific binding to sialic acid^46,47^. Non-B cells and dead cells were gated out with CD3, CD8, CD14, CD56, and LIVE/DEAD (Near-IR, ThermoFisher) staining. MBCs were gated on as CD19^+^, CD20^+^, and then further differentiated into CD27^+^CD21^+^ and CD27^+^CD21^lo^ populations. Antibodies used for antigen-specific memory B-cell assessment are listed in Supplementary Table 5. Statistical differences between mRNA-1010 and FLUAD influenza-specific B-cell responses were evaluated in GraphPad Prism using a two-way ANOVA with Šídák’s multiple comparisons test (*p* < 0.05).

### Assessment of HA-specific T-cell responses by intracellular cytokine staining

Antigen-specific T-cell responses were assessed in a subset of participants (*n* = 16 per vaccine group) on days 1, 15, and 29. Cryopreserved PBMCs were thawed and rested overnight. Cells were then stimulated with 2 µg/mL of the respective peptide pool (15-amino-acid peptides overlapping by 11 amino acids, spanning the whole length of the respective HA antigens, A/Wisconsin/588/2019 H1N1 HA, A/Darwin/6/2021 H3N2 HA, B/Austria/1359417/2021 HA, and B/Phuket/3073/2013 HA; Genscript Biotech Corp) in the presence of protein transport inhibitor, Fc block (BD), CD107a, and CD40 blocking antibody (Miltenyi) for 7.5 h. Dimethyl sulfoxide (DMSO) (final v/v at 0.5%) and cell stimulation cocktail (ThermoFisher) were used in place of peptide pools as negative and positive controls, respectively. Following incubation, cells were washed and stained for surface markers (fixable Live/Dead stain, CD45RA, CD8, CD14, CD16, CD19, CD56, CD4, CCR7, and TCRγδ). Cells were then fixed and permeabilized using the BD Cytofix/Cytoperm kit and stained intracellularly for CD3, IL-5, CD154 (CD40L), IFN-γ, IL-17A, TNF-α, IL-4, IL-13, IL-2, granzyme B, and CD69. After final washes, cells were resuspended in FC buffer containing 0.5% paraformaldehyde and analyzed on a five-laser Aurora Spectral Flow Cytometer (Cytek Biosciences). All antibodies are listed in Supplementary Table 6. Data analysis was performed using OMIQ (Insightful Science). FlowAI was applied to remove irregular events. Antigen-specific responses were determined by subtracting DMSO control values from peptide-stimulated conditions. Boolean gating was used to assess CD8^+^ T-cell subsets and CD4^+^ T-cell subsets based on cytokine expression. Polyfunctional CD8^+^ T cells were defined as those expressing IFN-γ, TNF-α, and/or IL-2, while CD4^+^ T cells were assessed for IFN-γ, TNF-α, IL-2, and/or CD40L. Statistical differences between mRNA-1010 and FLUAD in T-cell responses induced by flu antigens post-vaccination were evaluated in GraphPad Prism using a two-way ANOVA with Šídák’s multiple comparisons test (*p* < 0.05).

## Supplementary information

Supplementary Information

## Data Availability

As the trial is ongoing, access to patient-level data presented in the article and supporting clinical documents by qualified external researchers who provide methodologically sound scientific proposals may be available upon reasonable request for products or indications that have been approved by regulators in the relevant markets and subject to review from 24 months after study completion. Such requests can be made to Moderna, Inc., 325 Binney Street, Cambridge, MA 02142; data_sharing@modernatx.com. A materials transfer and/or data access agreement with the sponsor will be required for accessing shared data. All other relevant data are presented in the paper.
