# Supplementary material for: An mRNA influenza vaccine induces immunity comparable to an adjuvanted vaccine in a randomized trial
Source: NPJ Vaccines. 2026 Jan 14;11:50. doi: 10.1038/s41541-026-01370-7 (PMC12901179; doi:10.1038/s41541-026-01370-7)
Supplement: Supplementary file 1 — Supplementary Information [file 41541_2026_1370_MOESM1_ESM.pdf]

Supplementary Information

An mRNA influenza vaccine induces immunity comparable to an adjuvanted vaccine in a randomized trial

Carole Henry, Daniel Makrinos, Runxia Liu, Maria Cavallaro, Brooke Fenderson, Yanbo Sun, Xiaolin Chang, Eleanor Astley, Bethany Girard[1], Wen-Han Yu, Jaap Oostendorp, Anthony DiPiazza, Robert Paris

Supplementary Figure 1. Participant disposition (safety set).

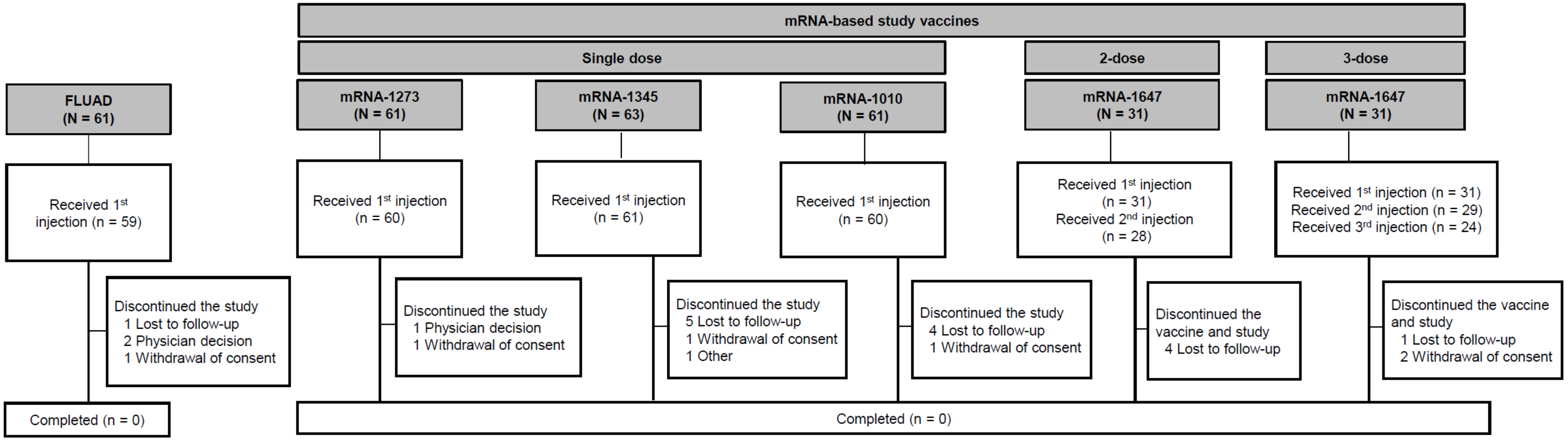

Supplementary Figure 2: Gating strategy used for antigen-specific Memory B Cells

Fluorescence-Activated Cell Sorting (FACS) gating strategy used to identify hemagglutinin-specific memory B cells. Initial gating was performed on lymphocytes (Forward Scatter [FSC] vs. SSC [Side Scatter]), singlets (Forward Scatter Area [FSC-A] vs. Forward Scatter Height [FSC-H]), live (exclusion of dead cells) non-monocyte/T/NK (CD14/3/8/56) and $CD19^{+}CD20^{+}$ B cells. Memory B cells were then gated as $CD27^{+}$ (or subdivided between $CD27^{+}CD21^{+}$ and $CD27^{+}CD21^{lo}$ populations) and antigen-specific using four hemagglutinin probes labeled with different fluorophores.

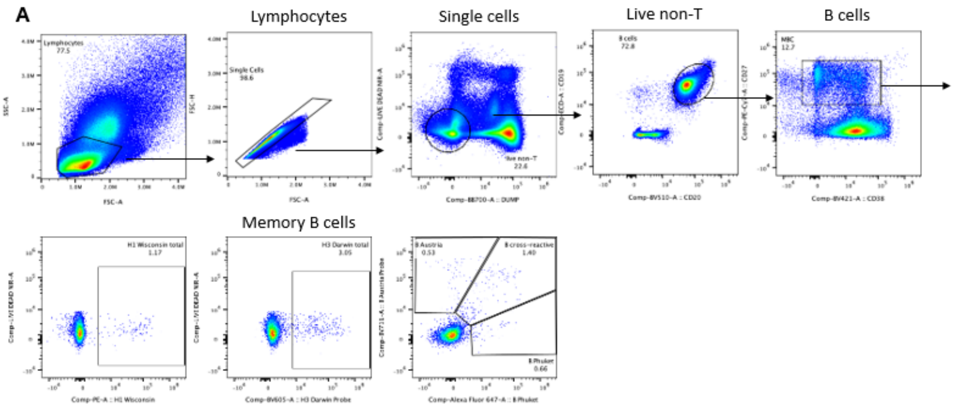

Supplementary Figure 3: Gating strategy used for T-cell intracellular staining

Fluorescence-Activated Cell Sorting (FACS) gating strategy used to identify cytokine-producing, activated, non-naïve $CD4^+$ and $CD8^+$ T cells following stimulation with the B/Phuket/3073/2013 HA antigen, as an example, or dimethyl sulfoxide (DMSO; negative control). Initial gating was performed on lymphocytes (Forward Scatter [FSC] vs. SSC [Side Scatter]), singlets (Forward Scatter Area [FSC-A] vs. Forward Scatter Height [FSC-H]), live (exclusion of dead cells), non-monocyte/B/NK (CD14/16/19/56), and $CD3^+$ T cells (CD3+ TCRgd-). Non-naïve T cells were selected by excluding $CD45RA^+CCR7^+$ populations, followed by gating on $CD4^+$ or $CD8^+$ T-cell subsets. Activated cytokine-producing cells were identified as $CD69^+$ and cytokine-positive (e.g., IFN-γ, TNF-α, IL-2).

Panel A represents $CD8^+$ T cells stimulated with DMSO as a negative control for background cytokine expression. Panel B shows $CD8^+$ T cells stimulated with the B/Phuket/3073/2013 HA antigen, highlighting antigen-specific cytokine responses. Panel C displays $CD4^+$ T cells stimulated with DMSO, serving as a baseline control, while panel D presents $CD4^+$ T cells stimulated with the B/Phuket/3073/2013 HA antigen, capturing the antigen-specific cytokine response. This gating strategy ensures the identification of antigen-specific, cytokine-producing, activated non-naïve T cells, distinguishing responses from background activation.

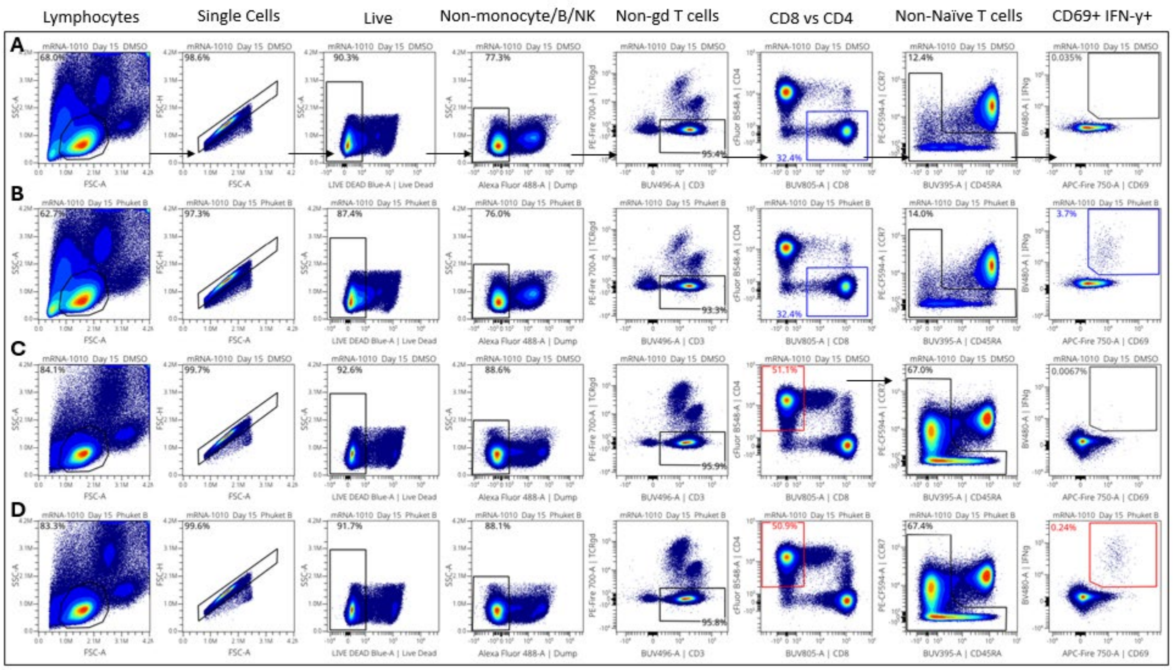

Supplementary Figure 4: CD8 and CD4 (Th1/Th2/Th17) cytokines

Intracellular cytokine staining of peripheral mononuclear blood cells (PBMCs) collected after vaccination with mRNA-1010 (*n* = 15) or FLUAD (*n* = 16) reveals strain-specific T-cell responses, displayed in panels A–C. PBMCs obtained on days 1 (baseline), 15, and 29 were stimulated with overlapping hemagglutinin peptide pools from A/Wisconsin, A/Darwin, B/Austria, and B/Phuket, and the frequency of non-naïve T cells producing effector molecules was quantified. Panel A depicts $CD8^+$ mediators (IFN-γ, TNF-α, IL-2, CD107a, granzyme B); panel B shows $CD4^+$ Th1 cytokines plus CD40L (IFN-γ, TNF-α, IL-2, CD40L); and panel C presents $CD4^+$ Th2/Th17 cytokines (IL-4, IL-5, IL-13, IL-17A). Box colors represent each specific mediator measured (legend right). For each strain, box-and-whisker plots display responses for mRNA-1010 (left) and FLUAD (right) at each time point, with boxes representing the interquartile range, horizontal bars the median, whiskers the 5th–95th percentiles, individual symbols the participants, and a dashed line marking the lower limit of detection (0.0025%). *Abbreviations*: HA, hemagglutinin; IFN, interferon; IL, interleukin; LLOD, lower limit of detection; PBMC, peripheral blood mononuclear cell; Th, T helper; TNF, tumor necrosis factor; ULOQ, upper limit of quantification.

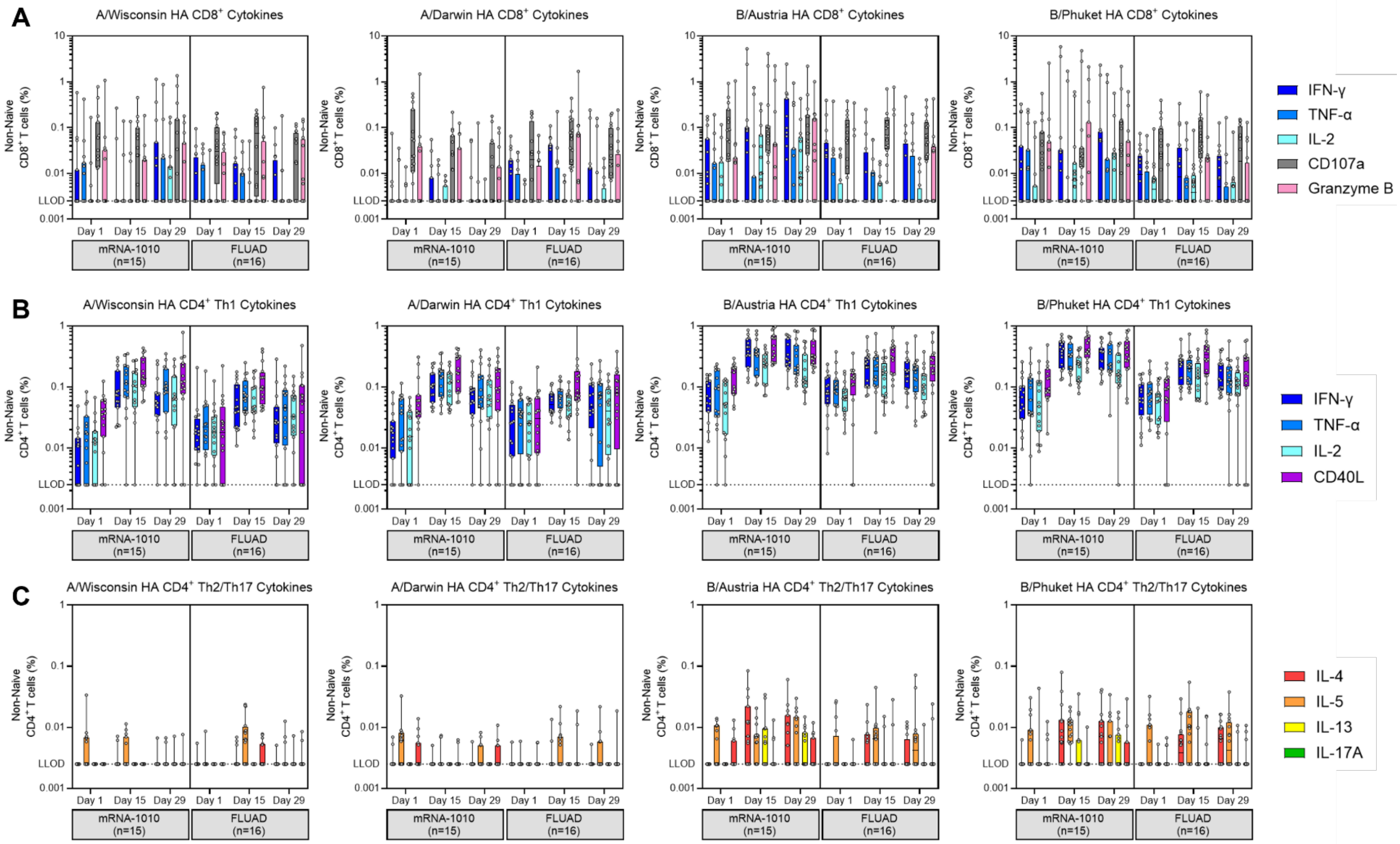
A
A/Wisconsin HA CD8+ Cytokines
A/Darwin HA CD8+ Cytokines
B/Austria HA CD8+ Cytokines
B/Phuket HA CD8+ Cytokines
Non-Naive CD8+ T cells (%)
IFN-γ
TNF-α
IL-2
CD107a
Granzyme B
B
A/Wisconsin HA CD4+ Th1 Cytokines
A/Darwin HA CD4+ Th1 Cytokines
B/Austria HA CD4+ Th1 Cytokines
B/Phuket HA CD4+ Th1 Cytokines
Non-Naive CD4+ T cells (%)
IFN-γ
TNF-α
IL-2
CD40L
C
A/Wisconsin HA CD4+ Th2/Th17 Cytokines
A/Darwin HA CD4+ Th2/Th17 Cytokines
B/Austria HA CD4+ Th2/Th17 Cytokines
B/Phuket HA CD4+ Th2/Th17 Cytokines
Non-Naive CD4+ T cells (%)
IL-4
IL-5
IL-13
IL-17A
LLOD
Day 1 Day 15 Day 29
mRNA-1010 (n=15)
FLUAD (n=16)

Supplementary Figure 4. The size exclusion chromatography profile of each evaluated construct

Trimeric recombinant HA with avi-tags were biotinylated using BirA biotin ligase enzyme kit (Avidity LLC). Size exclusion chromatography was performed using a Superdex 200 prepacked column (Cytiva) to remove excess biotin and purify for trimeric HA. *Abbreviations*: HA, hemagglutinin.

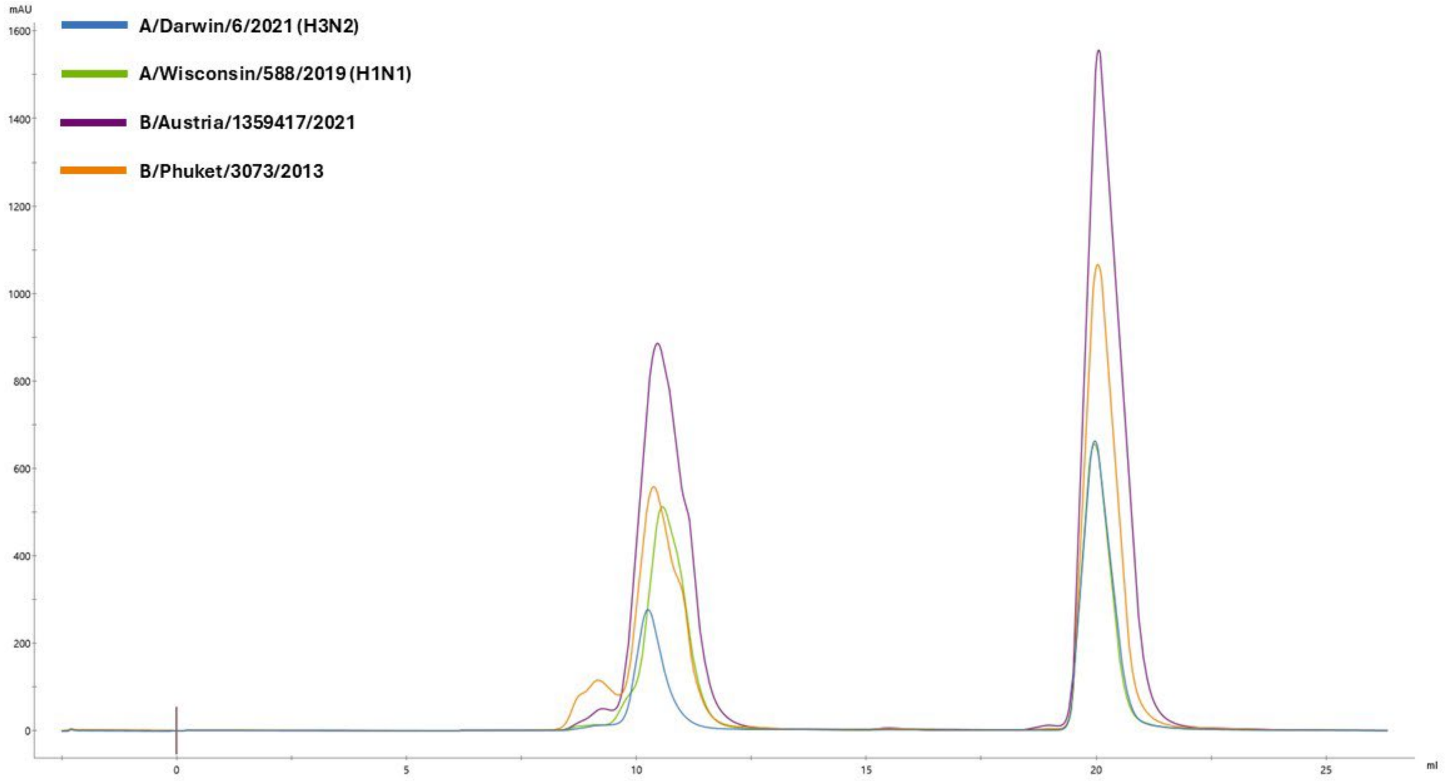

Supplementary Table 1. Summary of influenza antibody responses by HAI through Day 361 for all participants

	**mRNA-1010**				**FLUAD**			
	**A-H1N1**	**A-H3N2**	**B/Victoria**	**B/Yamagata**	**A-H1N1**	**A-H3N2**	**B/Victoria**	**B/Yamagata**
**Baseline (Day 1)**								
**n**	55	55	55	55	57	57	57	57
**GMT**<br>**(95% CI)**	41.0<br>(32.3-52.1)	27.4<br>(22.4-33.5)	65.9<br>(53.7-80.7)	57.3<br>(44.3-74.0)	41.4<br>(32.3- 53.2)	33.3<br>(26.7-41.6)	65.1<br>(54.6-77.6)	56.5<br>(42.1-75.9)
**Day 8**								
**n**	55	55	55	55	57	57	57	57
**GMT**<br>**(95% CI)**	144.0<br>(108.6-190.7)	75.8<br>(60.7-94.7)	90.4<br>(76.1-107.4)	117.2<br>(94.2-145.7)	170.6<br>(129.4- 224.9)	89.5<br>(71.9-111.4)	112.7<br>(95.2-133.5)	137.9<br>(111.3-170.8)
**GMFR**<br>**(95% CI)**	3.6<br>(2.7-4.7)	2.6<br>(2.1-3.2)	1.4<br>(1.2-1.6)	2.1<br>(1.7-2.6)	4.2<br>(3.2-5.5)	3.0<br>(2.4-3.8)	1.7<br>(1.5-2.0)	2.5<br>(2.0-3.0)
**SRR (%)**<br>**(95% CI)**	53<br>(39-66)	38<br>(25-52)	6<br>(1-15)	26<br>(15-39)	49<br>(36-63)	39<br>(26-52)	12<br>(5-24)	33<br>(21-47)
**Titer ≥ 1:40 (%)**<br>**(95% CI)**	89<br>(78-96)	80<br>(67-90)	95<br>(85-99)	89<br>(78-96)	93<br>(83-98)	91<br>(81-97)	100<br>(94-100)	93<br>(83-98)
**Day 15**								
**n**	55	55	55	55	57	57	57	57
**GMT**<br>**(95% CI)**	229.7<br>(176.1-299.5)	129.6<br>(102.8-163.5)	107.8<br>(88.0-132.1)	166.9<br>(135.9-204.9)	275.8<br>(212.5-357.9)	166.4<br>(132.5-209.2)	135.2<br>(110.8-165.1)	182.3<br>(149.1-223.0)
**GMFR**<br>**(95% CI)**	5.7<br>(4.3-7.4)	4.4<br>(3.5-5.5)	1.7<br>(1.4, 2.0)	3.0<br>(2.4-3.7)	6.8<br>(5.2-8.8)	5.6<br>(4.5-7.1)	2.1<br>(1.7-2.5)	3.3<br>(2.7-4.0)
**SRR (%)**<br>**(95% CI)**	67<br>(53-79)	56<br>(42-70)	16<br>(8-29)	46<br>(32-59)	63<br>(49-76)	63<br>(49-76)	25<br>(14-38)	46<br>(32-59)
**Titer ≥ 1:40 (%)**<br>**(95% CI)**	100<br>(94-100)	91<br>(80-97)	98<br>(90-100)	98<br>(90-100)	96<br>(88-100)	100<br>(94-100)	98<br>(91-100)	95<br>(85-99)
**Day 29**								
**n**	55	55	55	55	57	57	57	57
**GMT**<br>**(95% CI)**	199.9<br>(153.3- 260.5)	110.0<br>(86.3-140.2)	100.0<br>(82.3-121.4)	149.0<br>(121.1-183.4)	239.8<br>(184.9-311.1)	136.2<br>(107.3-173.0)	128.9<br>(106.5-156.0)	148.3<br>(120.9-181.8)
**GMFR**<br>**(95% CI)**	4.9<br>(3.8-6.4)	3.7<br>(2.9-4.8)	1.5<br>(1.3-1.9)	2.7<br>(2.2-3.3)	5.9<br>(4.6-7.7)	4.6<br>(3.6-5.9)	2.0<br>(1.6-2.4)	2.6<br>(2.2-3.2)
**SRR (%)**<br>**(95% CI)**	67<br>(53-79)	55<br>(41-68)	9<br>(3-20)	35<br>(22-49)	61<br>(48-74)	60<br>(46-72)	25<br>(14-38)	35<br>(23-49)
**Titer ≥ 1:40 (%)**<br>**(95% CI)**	96<br>(87-100)	87<br>(76-95)	98<br>(90-100)	95<br>(85-99)	96<br>(88-100)	98<br>(91-100)	98<br>(91-100)	96<br>(88-100)
**Day 91**								
**n**	52	52	52	52	54	54	54	54
**GMT**<br>**95% CI)**	128.4<br>(102.5-160.8)	89.3<br>(72.3-110.3)	102.1<br>(87.0-119.8)	134.7<br>(116.6-155.6)	135.1<br>(108.3-168.6)	105.4<br>(85.6-129.6)	111.5<br>(95.3-130.4)	141.4<br>(122.7-162.9)
**GMFR**<br>**(95% CI)**	3.2<br>(2.5-4.0)	3.0<br>(2.5-3.7)	1.6<br>(1.3-1.8)	2.4<br>(2.1-2.8)	3.3<br>(2.7-4.2)	3.6<br>(2.9-4.4)	1.7<br>(1.5-2.0)	2.5<br>(2.2-2.9)
**SRR (%)**<br>**(95% CI)**	44<br>(30-59)	44<br>(30-59)	10<br>(3-21)	42<br>(29-57)	50<br>(36-64)	52<br>(38-66)	20<br>(11-34)	39<br>(26-53)
**Titer ≥ 1:40 (%)**<br>**(95% CI)**	98<br>(90-100)	94<br>(84-99)	100<br>(93-100)	100<br>(93-100)	98<br>(90-100)	100<br>(93-100)	100<br>(93-100)	100<br>(93-100)

**Day 181**								
**n**	49	49	49	49	52	52	52	52
**GMT (95% CI)_**	100.5 (80.7-125.2)	82.5 (65.6-103.8)	93.0 (79.9-108.3)	120.4 (102.3-141.7)	107.8 (87.0-133.7)	82.9 (66.2-103.7)	102.8 (88.6-119.2)	126.0 (107.5-147.7)
**GMFR (95% CI)**	2.5 (2.0-3.1)	2.8 (2.2-3.5)	1.4 (1.2-1.7)	2.2 (1.8-2.5)	2.7 (2.1-3.3)	2.8 (2.2-3.5)	1.6 (1.4-1.8)	2.3 (1.9-2.6)
**SRR (%) (95% CI)**	29 (17-43)	33 (20-48)	12 (5-25)	27 (15-41)	48 (34-62)	42 (29-57)	15 (7-28)	37 (24-51)
**Titer ≥ 1:40 (%) (95% CI)**	94 (83-99)	88 (75-95)	96 (86-100)	98 (89-100)	90 (79-97)	94 (84-99)	100 (93-100)	100 (93-100)
**Day 361**								
**n**	44	44	44	44	45	45	45	45
**GMT (95% CI)**	77.4 (63.2-94.8)	50.0 (39.2-63.6)	98.1 (83.3-115.5)	73.0 (64.1-83.2)	84.0 (68.8-102.6)	47.5 (37.5-60.3)	90.3 (76.9-106.1)	72.6 (63.8-82.5)
**GMFR (95% CI)**	1.9 (1.6-2.3)	1.7 (1.3-2.2)	1.5 (1.3-1.8)	1.3 (1.1-1.5)	2.1 (1.7-2.5)	1.6 (1.3-2.1)	1.4 (1.2-1.6)	1.3 (1.1-1.5)
**SRR (%) (95% CI)**	21 (10-35)	16 (7-30)	7 (1-19)	9 (3-22)	31 (18-47)	20 (10-35)	7 (1-18)	18 (8-32)
**Titer ≥ 1:40 (%) (95% CI)**	89 (75-96)	57 (41-72)	98 (88-100)	100 (92-100)	87 (73-95)	71 (56-84)	100 (92-100)	98 (88-100)

CI, confidence interval; GMFR, geometric mean fold rise; GMT, geometric mean titer; SRR, seroresponse rate.

Supplementary Table 2. Summary of influenza antibody responses by HAI through Day 361 for participants 18-49 years of age

	**mRNA-1010**				**FLUAD**			
	**A-H1N1**	**A-H3N2**	**B/Victoria**	**B/Yamagata**	**A-H1N1**	**A-H3N2**	**B/Victoria**	**B/Yamagata**
**Baseline (Day 1)**								
**n**	29	29	29	29	27	27	27	27
**GMT**	40.5	31.5	54.0	79.0	47.8	33.8	66.9	99.5
**(95% CI)**	(30.6-53.6)	(23.5-42.1)	(45.2-64.6)	(57.7-108.2)	(33.3-68.7)	(23.6-48.6)	(50.2-89.0)	(66.8-148.0)
**Day 8**								
**n**	29	29	29	29	27	27	27	27
**GMT**	192.4	98.8	70.3	212.9	266.5	102.3	101.6	252.7
**(95% CI)**	(128.4-288.3)	(70.1-139.4)	(56.0-88.2)	(162.8-278.5)	(175.2-405.5)	(71.6-146.2)	(80.2-128.6)	(191.3-334.0)
**GMFR**	4.5	3.2	1.2	2.5	6.2	3.3	1.7	2.9
**(95% CI)**	(3.0-6.7)	(2.2-4.5)	(0.9-1.5)	(1.9-3.2)	(4.1-9.4)	(2.3-4.7)	(1.4-2.2)	(2.2-3.9)
**SRR (%)**	69	55	3	31	59	41	11	33
**(95% CI)**	(49-85)	(36-74)	(0-18)	(15-51)	(39-78)	(22-61)	(2-29)	(17-54)
**Titer ≥ 1:40 (%)**	93	83	93	100	100	93	100	100
**(95% CI)**	(77-99)	(64-94)	(77-99)	(88-100)	(87-100)	(76-99)	(87-100)	(87-100)
**Day 15**								
**n**	29	29	29	29	27	27	27	27
**GMT**	333.5	161.5	87.1	267.3	386.7	173.3	102.8	287.2
**(95% CI)**	(236.7-470.1)	(115.7-225.3)	(68.5-110.8)	(205.0-348.6)	(270.8-552.1)	(122.6-244.9)	(80.1-132.0)	(218.0-378.4)
**GMFR**	7.8	5.2	1.5	3.1	9.0	5.5	1.7	3.3
**(95% CI)**	(5.5-10.9)	(3.7-7.2)	(1.2-1.9)	(2.4-4.0)	(6.3-12.8)	(3.9-7.8)	(1.3-2.2)	(2.5-4.4)
**SRR (%)**	79	62	14	48	67	63	15	44
**(95% CI)**	(60-92)	(42-79)	(4-32)	(29-67)	(46-83)	(42-81)	(4-34)	(25-65)
**Titer ≥ 1:40 (%)**	100	97	100	100	100	100	96	96
**(95% CI)**	(88-100)	(82-100)	(88-100)	(88-100)	(87-100)	(87-100)	(81-100)	(81-100)
**Day 29**								
**n**	29	29	29	29	27	27	27	27
**GMT**	282.0	125.6	78.3	226.2	358.0	143.0	105.6	234.0
**(95% CI)**	(200.0-397.8)	(87.2-180.9)	(62.4-98.1)	(171.9-297.6)	(250.5-511.6)	(97.9-208.8)	(83.5-133.4)	(176.0-311.2)
**GMFR**	6.6	4.0	1.3	2.6	8.3	4.6	1.8	2.7
**(95% CI)**	(4.6-9.2)	(2.8-5.8)	(1.1-1.7)	(2.0-3.5)	(5.8-11.9)	(3.1-6.7)	(1.4-2.2)	(2.0-3.6)
**SRR (%)**	79	59	3	35	67	63	15	41
**(95% CI)**	(60-92)	(39-76)	(0-18)	(18-54)	(46-83)	(42-81)	(4-34)	(22-61)
**Titer ≥ 1:40 (%)**	100	90	100	97	100	96	96	100
**(95% CI)**	(88-100)	(73-98)	(88-100)	(82-100)	(87-100)	(81-100)	(81-100)	(87-100)
**Day 91**								
**n**	27	27	27	27	25	25	25	25
**GMT**	152.6	96.1	77.2	168.5	197.2	114.6	96.3	154.4
**(95% CI)**	(111.0-209.8)	(71.7-128.8)	(62.9-94.9)	(138.3-205.2)	(141.7-274.3)	(84.5-155.2)	(77.8-119.2)	(125.8-189.4)
**GMFR**	3.5	3.1	1.3	2.0	4.6	3.7	1.6	1.8
**(95% CI)**	(2.6-4.9)	(2.3-4.1)	(1.1-1.6)	(1.6-2.4)	(3.3-6.4)	(2.7-5.0)	(1.3-2.0)	(1.5-2.2)
**SRR (%)**	48	37	4	33	64	60	20	28
**(95% CI)**	(29-68)	(19-58)	(0-19)	(17-54)	(43-82)	(39-79)	(7-41)	(12-49)
**Titer ≥ 1:40 (%)**	100	96	100	100	100	100	100	100
**(95% CI)**	(87-100)	(81-100)	(87-100)	(87-100)	(86-100)	(86-100)	(86-100)	(86-100)

**Day 181**								
**n**	25	25	25	25	24	24	24	24
**GMT (95% CI)**	104.1 (77.6-139.7)	75.5 (57.1-99.8)	85.7 (70.6-103.9)	149.4 (119.7-186.3)	151.7 (112.0-205.6)	97.3 (73.0-129.8)	91.7 (75.2-111.8)	124.3 (99.0-156.0)
**GMFR (95% CI)**	2.4 (1.8-3.2)	2.4 (1.8-3.2)	1.4 (1.2-1.7)	1.7 (1.4-2.2)	3.5 (2.6-4.8)	3.1 (2.3-4.2)	1.5 (1.3-1.9)	1.4 (1.2-1.8)
**SRR (%) (95% CI)**	24 (9-45)	20 (7-41)	12 (3-31)	8 (1-26)	63 (41-81)	50 (29-71)	4 (0-21)	17 (5-37)
**Titer ≥ 1:40 (%) (95% CI)**	96 (80-100)	84 (64-95)	96 (80-100)	100 (86-100)	96 (79-100)	100 (86-100)	100 (86-100)	100 (86-100)
**Day 361**								
**n**	23	23	23	23	21	21	21	21
**GMT (95% CI)**	78.3 (58.6-104.8)	50.7 (36.7-70.1)	80.5 (65.9-98.3)	93.4 (76.3-114.2)	114.7 (84.8-155.2)	53.4 (38.1-74.8)	83.9 (68.0-103.4)	88.0 (71.3-108.5)
**GMFR (95% CI)**	1.8 (1.4-2.4)	1.6 (1.2-2.2)	1.4 (1.1-1.7)	1.1 (0.9-1.3)	2.7 (2.0-3.6)	1.7 (1.2-2.4)	1.4 (1.1-1.7)	1.0 (0.8-1.3)
**SRR (%) (95% CI)**	17 (5-39)	9 (1-28)	4 (0-22)	4 (0-22)	38 (18-62)	19 (5-42)	0 (0-16)	5 (0-24)
**Titer ≥ 1:40 (%) (95% CI)**	83 (61-95)	61 (39-80)	96 (78-100)	100 (85-100)	100 (84-100)	76 (53-92)	100 (84-100)	100 (84-100)

CI, confidence interval; GMFR, geometric mean fold rise; GMT, geometric mean titer; SRR, seroresponse rate.

Supplementary Table S3. Summary of influenza antibody responses by HAI through Day 361 for participants 50-75 years of age

	mRNA-1010				FLUAD			
	**A-H1N1**	**A-H3N2**	**B/Victoria**	**B/Yamagata**	**A-H1N1**	**A-H3N2**	**B/Victoria**	**B/Yamagata**
**Baseline (Day 1)**								
**n**	26	26	26	26	30	30	30	30
**GMT (95% CI)**	41.6 (27.3-63.3)	23.5 (17.6-31.2)	82.2 (56.2-120.1)	40.0 (27.1-59.0)	36.4 (25.5-52.1)	32.8 (24.6-43.8)	63.5 (50.5-79.9)	34.0 (23.8-48.6)
**Day 8**								
**n**	26	26	26	26	30	30	30	30
**GMT (95% CI)**	103.5 (72.4-147.8)	56.4 (42.7-74.5)	121.0 (93.3-156.9)	62.1 (47.2-81.6)	115.5 (82.9-161.0)	79.6 (61.5-103.2)	122.4 (96.1-155.9)	78.2 (60.7-100.9)
**GMFR (95% CI)**	2.7 (1.9-3.9)	2.0 (1.5-2.7)	1.7 (1.3-2.2)	1.7 (1.3-2.2)	3.0 (2.2-4.2)	2.9 (2.2-3.7)	1.7 (1.3-2.2)	2.1 (1.7-2.8)
**SRR (%) (95% CI)**	35 (17-56)	19 (7-39)	8 (1-25)	19 (7-39)	40 (23-59)	37 (20-56)	13 (4-31)	33 (17-53)
**Titer ≥ 1:40 (%) (95% CI)**	85 (65-96)	77 (56-91)	96 (80-100)	77 (56-91)	87 (69-96)	90 (73-98)	100 (88-100)	87 (69-96)
**Day 15**								
**n**	26	26	26	26	30	30	30	30
**GMT (95% CI)**	150.5 (104.0-217.9)	101.5 (72.9-141.1)	138.3 (99.8-191.6)	101.8 (76.6-135.1)	205.8 (145.9-290.4)	161.1 (118.4-219.0)	171.1 (126.3-231.8)	118.5 (91.1-154.3)
**GMFR (95% CI)**	3.9 (2.7-5.6)	3.6 (2.6-5.1)	1.9 (1.4-2.7)	2.8 (2.1-3.7)	5.4 (3.8-7.6)	5.8 (4.3-7.9)	2.4 (1.8-3.2)	3.2 (2.5-4.2)
**SRR (%) (95% CI)**	54 (33-73)	50 (30-70)	19 (7-39)	42 (23-63)	60 (41-77)	63 (44-80)	33 (17-53)	47 (28-66)
**Titer ≥ 1:40 (%) (95% CI)**	100 (87-100)	85 (65-96)	96 (80-100)	96 (80-100)	93 (78-99)	100 (88-100)	100 (88-100)	93 (78-99)
**Day 29**								
**n**	26	26	26	26	30	30	30	30
**GMT (95% CI)**	135.2 (93.5-195.5)	94.9 (67.9-132.5)	132.9 (96.8-182.4)	96.5 (72.3-128.7)	169.2 (120.0-238.4)	130.9 (95.9-178.6)	152.5 (113.5-204.7)	96.3 (73.6-125.9)
**GMFR (95% CI)**	3.5 (2.4-5.1)	3.4 (2.4-4.8)	1.9 (1.4-2.6)	2.6 (2.0-3.5)	4.4 (3.1-6.2)	4.7 (3.4-6.4)	2.1 (1.6-2.9)	2.6 (2.0-3.4)
**SRR (%) (95% CI)**	54 (33-73)	50 (30-70)	15 (4-35)	35 (17-56)	57 (37-75)	57 (37-75)	33 (17-53)	30 (15-49)
**Titer ≥ 1:40 (%) (95% CI)**	92 (75-99)	85 (65-96)	96 (80-100)	92 (75-99)	93 (78-99)	100 (88-100)	100 (88-100)	93 (78-99)
**Day 91**								
**n**	25	25	25	25	29	29	29	29
**GMT (95% CI)**	104.8 (78.0-140.9)	82.6 (60.1-113.3)	139.9 (110.8-176.7)	109.6 (89.3-134.4)	97.9 (74.4-128.9)	98.4 (73.2-132.1)	126.2 (101.6-156.8)	126.1 (104.4-152.5)
**GMFR (95% CI)**	2.7 (2.0-3.7)	3.0 (2.2-4.1)	2.0 (1.6-2.5)	3.0 (2.4-3.7)	2.6 (1.9-3.4)	3.5 (2.6-4.7)	1.8 (1.4-2.2)	3.4 (2.8-4.2)
**SRR (%) (95% CI)**	40 (21-61)	52 (31-72)	16 (5-36)	52 (31-72)	38 (21-58)	45 (26-64)	21 (8-40)	48 (29-67)
**Titer ≥ 1:40 (%) (95% CI)**	96 (80-100)	92 (74-99)	100 (86-100)	100 (86-100)	97 (82-100)	100 (88-100)	100 (88-100)	100 (88-100)

**Day 181**								
**n**	24	24	24	24	28	28	28	28
**GMT (95% CI)**	94.0 (68.5-129.1)	91.0 (62.8-131.8)	103.8 (81.5-132.2)	98.5 (78.0-124.6)	79.6 (59.4-106.8)	71.5 (50.6-100.9)	113.2 (90.5-141.6)	123.1 (99.0-152.9)
**GMFR (95% CI)**	2.5 (1.8-3.4)	3.3 (2.3-4.7)	1.5 (1.1-1.9)	2.7 (2.1-3.4)	2.1 (1.6-2.8)	2.6 (1.8-3.6)	1.6 (1.3-2.0)	3.4 (2.7-4.2)
**SRR (%) (95% CI)**	33 (16-55)	46 (26-67)	13 (3-32)	46 (26-67)	36 (19-56)	36 (19-56)	25 (11-45)	54 (34-72)
**Titer ≥ 1:40 (%) (95% CI)**	92 (73-99)	92 (73-99)	96 (79-100)	96 (79-100)	86 (67-96)	89 (72-98)	100 (88-100)	100 (88-100)
**Day 361**								
**n**	21	21	21	21	24	24	24	24
**GMT (95% CI)**	75.5 (57.0-100.0)	49.9 (34.0-73.3)	122.8 (93.7-160.8)	56.8 (48.2-66.9)	64.7 (49.7-84.1)	43.2 (30.2-61.8)	95.4 (74.1-122.8)	59.4 (51.0-69.3)
**GMFR (95% CI)**	2.0 (1.5-2.6)	1.8 (1.2-2.6)	1.7 (1.3-2.3)	1.6 (1.3-1.8)	1.7 (1.3-2.2)	1.6 (1.1-2.2)	1.3 (1.0-1.7)	1.6 (1.4-1.9)
**SRR (%) (95% CI)**	24 (8-47)	24 (8-47)	10 (1-30)	14 (3-36)	25 (10-47)	21 (7-42)	13 (3-32)	29 (13-51)
**Titer ≥ 1:40 (%) (95% CI)**	95 (76-100)	52 (30-74)	100 (84-100)	100 (84-100)	75 (53-90)	67 (45-84)	100 (86-100)	96 (79-100)

CI, confidence interval; GMFR, geometric mean fold rise; GMT, geometric mean titer; SRR, seroresponse rate.

Supplementary Table 4. Constructs and yields for the assessment of antigen-specific memory B cells

Construct	Expected MW (kDa)	Concentration (mg/ml)	Final Yield (mg)
H1_Wisconsin_2019_ Fd-AVI-Th-H6-St	66.03	0.71	2.13
H3_Darwin/2021_Fd-AVI-Th-H6-St	66.36	0.40	0.90
HB_Austria_Fd-AVI-Th-H6-St	66.13	1.62	4.86
HB_Phuket_2013_Fd-AVI-Th-H6-St	66.27	0.85	3.13

MW, molecular weight.

Supplementary Table 5. Antibodies used for antigen-specific memory B cell assessment

Marker	Clone	Fluorochrome	Vendor	Catalog #
Live/Dead NIR	-	NIR	ThermoFisher	L34994
Human Fc block	Fc1	-	BD	564220
CD11c	3.9	BUV395	BD	748289
CD10	MEM-78	BUV496	BD	750190
CD71	L01.1	BUV661	BD	749818
CD24	ML5	BUV737	BD	741831
CD38	HIT2	BV421	BD	562444
CD20	2H7	BV510	Biolegend	302340
IgM	G20-127	BV786	BD Optibuild	740998
IgA	IS11-8E10	FITC	Miltenyi	130-113-475
CD3	OKT3	BB700	BD	566818
CD8	RPA-T8	BB700	BD	566452
CD14	M5E2	BB700	BD Optibuild	745790
CD56	B159	BB700	BD	566400
CD19	J3-119	ECD	Beckman	IM2708U
IgD	IA6-2	PE-Cy5	Biolegend	348250
CD27	323	PE-Cy7	Biolegend	302838
IgG	G18-145	APC	BD	550931
CD21	Bu32	Alexa Fluor 700	Biolegend	354918
Streptavidin	-	PE	Biolegend	405204
Streptavidin	-	BV605	Biolegend	405229
Streptavidin	-	BV711	Biolegend	405241
Streptavidin	-	Alexa Fluor 647	Biolegend	405237

Supplementary Table 6. Antibodies used for T-cell ICS

Marker	Clone	Fluorochrome	Vendor	Catalog #	RRID
CD107a	H4A3	PE	Biolegend	328608	AB_1186040
CD45RA	HI100	BUV395	BD	740298	AB_2740037
CD8	SK1	BUV805	BD	612890	AB_2870178
CD14	63D3	Alexa Fluor 488	Biolegend	367130	AB_2721360
CD16	3G8	Alexa Fluor 488	Biolegend	302019	AB_492974
CD19	HIB19	Alexa Fluor 488	Biolegend	302219	AB_389313
CD56	5.1H11	Alexa Fluor 488	Biolegend	362518	AB_2564093
CD4	SK3	cFluor B548	Cytek	R7-20043	AB_3076337
CCR7/CD197	150503	PE-CF594	BD	562381	AB_11153301
TCRɣδ	B1	PE-Fire 700	Biolegend	331238	AB_2876638
CD3	SK7	BUV496	BD	741206	AB_2870764
IL-5	TRFK5	BV421	Biolegend	504311	AB_2563161
CD154/CD40L	24-31	Pacific Blue	Biolegend	310820	AB_830699
IFNɣ	B27	BV480	BD	566100	AB_2739503
IL-17A	BL168	BV605	Biolegend	512326	AB_2563887
TNFa	Mab11	BV750	BD	566359	AB_2739709
IL-4	MP4-25D2	BV786	BD	564113	AB_2738601
IL-13	JES10-5A2	PE-Cy7	Biolegend	501914	AB_2616746
IL-2	MQ1-17H12	Alexa Fluor 647	Biolegend	500315	AB_493370
Granzyme B	GB11	R718	BD	566964	AB_2869975
CD69	FN50	APC/Fire 750	Biolegend	310946	AB_2616709
anti-CD40 Hu antibody	HB14	-	Miltenyi	130-094-133	AB_10839704
Human BD Fc block	Fc1	-	BD	564220	AB_2869554
NA/LE human BD Fc block	Fc1	-	BD	564765	AB_2869612

Section/topic	No	CONSORT 2025 checklist item description	Reported on page no.
**Title and abstract**			
Title and structured abstract	1a	Identification as a randomised trial	2
	1b	Structured summary of the trial design, methods, results, and conclusions	2 (note: no subheadings are permitted)
**Open science**			
Trial registration	2	Name of trial registry, identifying number (with URL) and date of registration	2; 13
Protocol and statistical analysis plan	3	Where the trial protocol and statistical analysis plan can be accessed	18
Data sharing	4	Where and how the individual de-identified participant data (including data dictionary), statistical code and any other materials can be accessed	18
Funding and conflicts of interest	5a	Sources of funding and other support (eg, supply of drugs), and role of funders in the design, conduct, analysis and reporting of the trial	18
	5b	Financial and other conflicts of interest of the manuscript authors	19
**Introduction**			
Background and rationale	6	Scientific background and rationale	3-4
Objectives	7	Specific objectives related to benefits and harms	3-4

**Methods**			
Patient and public involvement	8	Details of patient or public involvement in the design, conduct and reporting of the trial	NA
Trial design	9	Description of trial design including type of trial (eg, parallel group, crossover), allocation ratio, and framework (eg, superiority, equivalence, non-inferiority, exploratory)	13-14; Supplemetary Fig. 1
Changes to trial protocol	10	Important changes to the trial after it commenced including any outcomes or analyses that were not prespecified, with reason	NA
Trial setting	11	Settings (eg, community, hospital) and locations (eg, countries, sites) where the trial was conducted	13-14
Eligibility criteria	12a	Eligibility criteria for participants	13-14
	12b	If applicable, eligibility criteria for sites and for individuals delivering the interventions (eg, surgeons, physiotherapists)	NA
Intervention and comparator	13	Intervention and comparator with sufficient details to allow replication. If relevant, where additional materials describing the intervention and comparator (eg, intervention manual) can be accessed	13-14
Outcomes	14	Prespecified primary and secondary outcomes, including the specific measurement variable (eg, systolic blood pressure), analysis metric (eg, change from baseline, final value, time to event), method of aggregation (eg, median, proportion), and time point for each outcome	13-14
Harms	15	How harms were defined and assessed (eg, systematically, non-systematically)	NA
Sample size	16a	How sample size was determined, including all assumptions supporting the sample size calculation	13; Supplementar y Fig 1
	16b	Explanation of any interim analyses and stopping guidelines	NA

Randomisation:			
Sequence generation	17a	Who generated the random allocation sequence and the method used	NA
	17b	Type of randomisation and details of any restriction (eg, stratification, blocking and block size)	13
			**Reported on page no.**
Allocation concealment mechanism	18	Mechanism used to implement the random allocation sequence (eg, central computer/telephone; sequentially numbered, opaque, sealed containers), describing any steps to conceal the sequence until interventions were assigned	NA
Implementation	19	Whether the personnel who enrolled and those who assigned participants to the interventions had access to the random allocation sequence	NA
Blinding	20a	Who was blinded after assignment to interventions (eg, participants, care providers, outcome assessors, data analysts)	NA
	20b	If blinded, how blinding was achieved and description of the similarity of interventions	
Statistical methods	21a	Statistical methods used to compare groups for primary and secondary outcomes, including harms	15-17
	21b	Definition of who is included in each analysis (eg, all randomised participants), and in which group	13-14; Supplementary Fig 1
	21c	How missing data were handled in the analysis	NA
	21d	Methods for any additional analyses (eg, subgroup and sensitivity analyses), distinguishing prespecified from post hoc	NA
**Results**			

Participant flow, including flow diagram	22a	For each group, the numbers of participants who were randomly assigned, received intended intervention, and were analysed for the primary outcome	13-14; Supplementary Fig 1
	22b	For each group, losses and exclusions after randomisation, together with reasons	13-14
Recruitment	23a	Dates defining the periods of recruitment and follow-up for outcomes of benefits and harms	13-14
	23b	If relevant, why the trial ended or was stopped	NA
Intervention and comparator delivery	24a	Intervention and comparator as they were actually administered (eg, where appropriate, who delivered the intervention/comparator, how participants adhered, whether they were delivered as intended (fidelity))	13-14
	24b	Concomitant care received during the trial for each group	NA
Baseline data	25	A table showing baseline demographic and clinical characteristics for each group	NA
Numbers analysed, outcomes and estimation	26	For each primary and secondary outcome, by group:	13-14
		● the number of participants included in the analysis	13-14
		● the number of participants with available data at the outcome time point	13-14
		● result for each group, and the estimated effect size and its precision (such as 95% confidence interval)	NA
		● for binary outcomes, presentation of both absolute and relative effect size	
Harms	27	All harms or unintended events in each group	NA
Ancillary analyses	28	Any other analyses performed, including subgroup and sensitivity analyses, distinguishing pre-specified from post hoc	NA
**Discussion**			
Interpretation	29	Interpretation consistent with results, balancing benefits and harms, and considering other relevant evidence	7-13
Limitations	30	Trial limitations, addressing sources of potential bias, imprecision, generalisability, and, if relevant, multiplicity of analyses	NA

Citation: Hopewell S, Chan AW, Collins GS, Hróbjartsson A, Moher D, Schulz KF, et al. CONSORT 2025 Statement: updated guideline for reporting randomised trials. BMJ. 2025; 388:e081123. https://dx.doi.org/10.1136/bmj-2024-081123
© 2025 Hopewell et al. This is an Open Access article distributed under the terms of the Creative Commons Attribution License (https://creativecommons.org/licenses/by/4.0/), which permits unrestricted use, distribution, and reproduction in any medium, provided the original work is properly cited.

*We strongly recommend reading this statement in conjunction with the CONSORT 2025 Explanation and Elaboration and/or the CONSORT 2025 Expanded Checklist for important clarifications on all the items. We also recommend reading relevant CONSORT extensions. See www.consort-spirit.org.
